# Combined bailing capsule and conventional therapies in the treatment of chronic renal failure: a meta-analysis and economic evaluation

**DOI:** 10.3389/fmed.2025.1609311

**Published:** 2025-06-25

**Authors:** Yumei He, Wei Li, He Zhu, Sheng Han

**Affiliations:** ^1^International Research Center for Medicinal Administration, Peking University, Beijing, China; ^2^School of Pharmaceutical Sciences, Peking University, Beijing, China

**Keywords:** chronic renal failure, Bailing capsules, Markov model, cost-effectiveness analysis, Chinese population

## Abstract

**Introduction:**

Bailing capsules are currently recommended for improving renal function in patients with chronic renal failure (CRF) in China. However, limited research assesses the clinical benefits of Bailing capsules in the context of healthcare resource utilization. Therefore, we conducted this study to compare the efficacy of Bailing capsules combined with conventional therapies and to assess their economic value from the Chinese healthcare system perspective.

**Methods:**

For the meta-analysis, six bibliographic databases were systematically searched for eligible randomized controlled trials (RCTs) from their inception until May 2025. For the economic evaluation, a Markov model was established to simulate the disease progression of patients over 20 years. The incremental cost-effectiveness ratio (ICER) was measured, and one-way and probabilistic sensitivity analyses were performed to observe model stability.

**Results:**

The use of Bailing capsules combined with conventional therapies was associated with a significant reduction in serum creatinine compared to conventional therapies alone (weighted mean difference [WMD]: –36.73 μmol/L; 95% confidence interval [CI]: –45.06 to –28.40; *p* < 0.001, *n* = 1,380 patients from 17 RCTs). Moreover, Bailing capsules combined with conventional therapies were associated with lower blood urea nitrogen (WMD: –2.52 mmol/L; 95% CI: –3.83 to –1.22; *p* < 0.001) and 24 h urinary protein (WMD: –0.39 g/L; 95% CI: –0.47 to –0.30; *p* < 0.001) levels than conventional therapy alone. However, no significant difference existed between Bailing capsules combined with conventional therapies and conventional therapy alone in terms of creatinine clearance rate (WMD: 4.81 mL/min; 95% CI: –0.45 to 10.06; *p* = 0.073). In the economic evaluation, combination therapy yielded 0.92 additional quality-adjusted life-years (QALYs) and incurred additional costs of Chinese Yuan (CNY) 21,335 over a 20-year horizon, resulting in an ICER of CNY 23,312 per QALY gained. This ICER was below China’s willingness-to-pay threshold of CNY 85,698 (2022 gross domestic product per capita). Sensitivity analyses confirmed the robustness of the results, with the combination therapy showing a 94% probability of cost-effectiveness at the threshold (probabilistic sensitivity analysis).

**Discussion:**

Bailing capsules combined with conventional therapies are associated with a greater reduction in serum creatinine and are likely to be cost-effective for patients with CRF in China.

## Introduction

Chronic renal failure (CRF) is a progressive loss of kidney function that occurs in various chronic kidney diseases (CKD). It is characterized by insufficient renal function, metabolite retention, and dysregulation of the internal environment (1, 2). The annual incidence of CRF in China has been increasing by approximately 0.3%. Consequently, the prevalence of CRF has been increasing (3, 4). The clinical manifestations of CRF include symptoms related to metabolic abnormalities and a systemic, multisystem inflammatory syndrome. Notably, patients with stage I–III CRF may be asymptomatic or present with moderate symptoms (5). However, severe symptoms of CRF include heart failure, gastrointestinal bleeding, and neurological disorders, which pose a lethal threat to patient health (6). Therefore, identifying effective treatments is essential to improve the prognosis of CRF.

Renal replacement therapy or kidney transplantation is typically performed for end-stage CRF; however, these options are expensive. Although the medical costs of non-dialysis patients with CRF are much lower than those of patients undergoing hemodialysis, conventional therapy is required to control blood pressure, glucose levels, and proteinuria (7). The Bailing capsule, composed of *Cordyceps* mycelia, is widely used to treat renal disease because it contains a variety of trace elements, amino acids, adenosine, and cordycepin. These elements improve blood circulation, inhibit neurotransmitter release, and regulate adenylate cyclase activity (8, 9). Studies have demonstrated that Bailing capsules can improve human immune function and reduce renal tubular epithelial cell damage, renal tubular atrophy, and overall kidney damage (10–12). Moreover, the use of Bailing capsules is associated with lower urea nitrogen levels and reduced proteinuria, which may improve the prognosis of CRF (13, 14). Recent meta-analyses have evaluated the efficacy of traditional Chinese medicine (TCM) in the management of CKD. A 2024 meta-analysis by Zheng et al. encompassing 66 studies, evaluated the efficacy of combining TCM with Western medicine for treating diabetic nephropathy. The analysis revealed that integrating TCM decoctions with Western medicine enhanced clinical effectiveness and yielded superior therapeutic outcomes compared to Western medicine alone, with no significant safety concerns identified (15). Another study evaluated Uremic Clearance Granules for CKD stages 3–5, showing Uremic Clearance Granules significantly reduce serum creatinine, triglyceride, and total cholesterol levels, while increasing glomerular filtration rate (GFR) and hemoglobin levels. It also effectively ameliorated signs and symptoms in patients with CKD (16). A 2025 study conducted by Wu et al. demonstrated that *Cordyceps* sinensis can be considered a dependable clinical treatment for patients with renal dysfunction (17). Another 2024 meta-analysis conducted by Tao et al. suggested that Bailing capsules were associated with lower levels of 24 h urinary protein, blood urea nitrogen (BUN), serum creatinine, and inflammatory markers. Network pharmacology analysis indicated that the Bailing capsule shares 190 common targets with CKD, with pharmacological mechanisms potentially related to immune response, inflammatory response, vascular endothelial damage, cell proliferation, and fibrosis (18). However, these analyses often focus solely on clinical efficacy, with limited evaluation of cost-effectiveness from the healthcare system’s perspective. Our study addresses this gap by combining clinical outcomes with a Markov model-based economic evaluation of Bailing capsules for CKD stages 3–5. Therefore, this study aimed to investigate the efficacy of Bailing capsules combined with conventional therapies for CRF treatment and to explore their economic value from the perspective of the Chinese healthcare system.

## Materials and methods

### Meta-analysis

#### Literature search and selection criteria

This study was conducted in accordance with the Preferred Reporting Items for Systematic Reviews and Meta-Analyses guidelines (19). The study was registered in International Platform of Registered Systematic Reviews and Meta-Analyses (INPLASY202550057). Randomized controlled trials (RCTs) comparing the efficacy of Bailing capsules combined with conventional therapy versus conventional therapy alone for CRF were included in this study. Publication language and status were not restricted. We systematically searched PubMed, Embase, Cochrane Library, China Science and Technology Journal Database, Wanfang, and China National Knowledge Internet databases to identify eligible RCTs from their inception through May 2025. We used “chronic renal failure,” “Bailing capsule,” and “randomized controlled trials” as search terms. Supplemental Table 1 shows details of the PubMed search strategy. Despite comprehensive searches of international databases, including PubMed and Embase, no additional eligible English-language RCTs that met our inclusion criteria were identified. This may be because Bailing capsules are predominantly used within the Chinese healthcare system, and most studies on their therapeutic efficacy for CRF have been conducted in China. We also searched for completed but unpublished trials on the ClinicalTrials.gov website. Additional eligible RCTs were identified by manually reviewing the reference lists of retrieved articles.

**TABLE 1:** Baseline characteristics of included studies.

Study	Region	Sample size (T/C)	Age (years, T/C)	Sex (male/female)		Disease duration (years, T/C)	Intervention		Dose of Bailing capsule	Treatment duration
				T	C		T	C		
Zheng et al. (37)	Shanghai	120 (60/60)	53.3 (54.0/52.5)	24/36	32/28	4.56 (5.89/3.23)	Bailing capsule plus conventional therapies	Conventional therapies	3 tablets × 3 times/day	3.0 months
Pu (38)	Guangdong	68 (34/34)	45.5 (46.5/44.5)	19/15	18/16	–	Bailing capsule plus alprostadil	Alprostadil	3 g × 3 times/day	3.0 months
Zheng and Wang (39)	Guangdong	72 (36/36)	61.5 (62.4/60.5)	22/14	20/16	4.41 (4.56/4.25)	Bailing capsule, conventional therapies, α-ketoacid	Conventional therapies, α-ketoacid	4 tablets × 3 times/day	12 weeks
Xiong et al. (40)	Guangxi	100 (50/50)	50.2 (50.5/49.9)	28/22	29/21	2.43 (2.46/2.40)	Bailing capsule plus conventional therapies	Conventional therapies	2 g × 3 times/day	12 weeks
Zhang and Wang (41)	Hubei	150 (75/75)	42.1 (41.0/43.2)	42/33	40/35	3.75 (3.64/3.86)	Bailing capsule plus conventional therapies	Conventional therapies	5 tablets × 3 times/day	3.0 months
Zheng et al. (42)	Shanghai	60 (30/30)	43.3 (44.0/42.5)	14/16	12/18	5.06 (5.89/4.23)	Bailing capsule plus conventional therapies	Conventional therapies	3 tablets × 3 times/day	3.0 months
Meng and Guo (43)	Hebei	68 (34/34)	46.6 (46.2/46.9)	19/15	18/16	3.30 (3.40/3.20)	Bailing capsule plus alprostadil	Alprostadil	2 g × 1 time/day	3.0 months
Ru (44)	Henan	110 (55/55)	54.8 (55.2/54.3)	30/25	32/23	–	Bailing capsule, conventional therapies, irbesartan	Conventional therapies, irbesartan	1 g × 3 times/day	24 weeks
Huang et al. (45)	Guangxi	64 (34/30)	45.0 (44.8/45.2)	18/14	14/16	3.85 (4.20/3.50)	Bailing capsule plus alprostadil	Alprostadil	2 g × 3 times/day	3.0 months
Yi (46)	Shandong	104 (52/52)	48.8 (48.3/49.2)	29/23	28/14	3.05 (3.10/3.00)	Bailing capsule, conventional therapies, α-ketoacid	Conventional therapies, α-ketoacid	1 g × 3 times/day	3.0 months
He (47)	Shanxi	80 (40/40)	63.0 (65.2/60.8)	19/21	25/15	–	Bailing capsule, conventional therapies, α-ketoacid	Conventional therapies, α-ketoacid	4 tablets × 3 times/day	1.0 year
Jiang (48)	Liaoning	38 (19/19)	46.1 (46.5/45.7)	9/10	11/8	–	Bailing capsule plus conventional therapies	Conventional therapies	5 tablets × 3 times/day	2.0 months
Shen and Zhang (49)	Guizhou	80 (40/40)	45.4 (45.6/45.2)	23/17	22/18	6.65 (6.50/6.80)	Bailing capsule plus conventional therapies	Conventional therapies	2 tablets × 3 times/day	24 weeks
Pan (50)	Shenzhen	64 (32/32)	43.4 (44.1/42.6)	19/13	20/12	–	Bailing capsule plus conventional therapies	Conventional therapies	5 tablets × 3 times/day	2.0 months
Wang and Jiang (51)	Zhejiang	80 (40/40)	60.9 (60.6/61.2)	26/14	29/11	–	Bailing capsule plus valsartan	Valsartan	1 g × 3 times/day	24 weeks
Hu (52)	Zhejiang	62 (31/31)	52.6 (52.6/52.5)	18/13	19/12	3.75 (3.70/3.80)	Bailing capsule plus alprostadil	Alprostadil	2 g × 3 times/day	2.0 weeks
Ren (53)	Heilongjiang	60 (30/30)	68.9 (68.8/68.9)	20/10	19/11	7.55 (7.50/7.60)	Bailing capsule plus pancreatic kinionoge	Pancreatic kinionoge	0.8 g × 3 times/day	8.0 weeks

Two reviewers (Yumei He and Wei Li) independently conducted the literature search and study selection using a standardized flow. Conflicts between the reviewers were resolved by an additional reviewer (Sheng Han) until a consensus was reached. The inclusion criteria were as follows: (1) non-dialysis patients with CRF; (2) intervention: Bailing capsule combined with conventional therapies; (3) control: conventional therapies alone; (4) outcome: serum creatinine, BUN, creatinine clearance rate (Ccr), and 24 h urinary protein; and (5) study design: RCT design. Studies were excluded if they met any of the following criteria: (1) the intervention included only artificial cordyceps without mention of Bailing capsules; (2) the treatment regimen contained other TCMs; or (3) baseline characteristics, treatment duration, and investigated outcomes were not reported.

#### Data extraction and quality assessment

Two reviewers (Yumei He and He Zhu) used a standardized flow chart to extract all relevant information from the included studies, including the first author’s surname, study region, sample size, cohort age and sex, disease duration, intervention, Bailing capsule dose, treatment duration, and reported outcomes. Study quality was assessed using the Risk of Bias 2 (ROB2) tool according to the methods described by the Cochrane Collaboration, including the randomization process, deviations from intended interventions, missing outcome data, measurement of outcomes, and selection of reported results (20). Data collection and quality assessment of individual trials were independently conducted by two reviewers (Yumei He and He Zhu), with any disagreements resolved by an additional reviewer (Sheng Han). The Grading of Recommendations Assessment, Development, and Evaluation (GRADE) approach was used to assess the certainty of evidence for each outcome (21).

#### Statistical analysis

The efficacy of the Bailing capsule was assessed based on the serum creatinine levels and pooled effect estimates. Heterogeneity among the included studies was evaluated using the I^2^ and Q statistics (22). A fixed-effects model was used to calculate the pooled effect estimate when p > 0.10 and I^2^ < 25%, while a random-effects model was applied if p < 0.10 or I^2^ > 25% (23). Sensitivity analysis was performed to assess the robustness of the pooled conclusion by sequentially removing individual trials (24). Subgroup analyses were performed to explore the potential heterogeneity in serum creatinine levels. Publication bias was assessed using funnel plots as well as Egger’s and Begg’s tests (25, 26). All statistical analyses were performed using Stata Statistical Software (version 15.0; StataCorp, College Station, TX, USA).

### Economic evaluation

#### Patients and regimens

A hypothetical cohort matching the inclusion criteria of the meta-analysis was included in the model. The treatment strategies included in the economic evaluation were as follows: (1) conventional therapy group: conventional therapies alone, aimed at stabilizing acid-base imbalance, reducing creatinine levels, protecting kidney function, and improving calcium and phosphorus metabolism disorders, with no restrictions on treatment drugs or dosages; (2) Bailing capsules group: use of Bailing capsules (2 g, three times daily) in addition to conventional treatment.

#### Model structure

A Markov model was created using Microsoft Excel that followed the progression of CKD (Supplementary Figure 1). The disease states in the model were segregated into CKD stages 3, 4, 5 (non-hemodialysis), 6 (hemodialysis), and death; CKD progression was irreversible. Considering that 89.1% of patients with end-stage renal disease undergo hemodialysis, this treatment was included in this study (27). Moreover, treatment with Bailing capsules mainly slowed CKD progression from stage 3 to stage 4 and from stage 4 to stage 5, with no significant effect on patients at CKD stage 5 or those undergoing hemodialysis. Additionally, treatment with Bailing capsules did not significantly affect CKD stages 3 and 4.

The model had a 20-year time horizon with a 1-year cycle length and a half-cycle correction applied to all costs and outcomes. We conducted an economic evaluation from the perspective of the Chinese healthcare system. Outcomes were measured as gains in quality-adjusted life-years (QALYs) and total direct medical costs. The incremental cost-effectiveness ratio (ICER) was also calculated. Costs and benefits were discounted at 5.0% annually in accordance with the China Guidelines for Pharmacoeconomic Evaluations (28). The gross domestic product (GDP) per capita in 2022 [Chinese Yuan (CNY) 85,698] was used as the willingness-to-pay (WTP) threshold.

#### Model inputs

Transition probabilities between various CKD stages were calculated using a microsimulation approach. GFR is the main predictor of renal function; however, it remains low in patients receiving appropriate treatments (29–32). Transition probabilities of various CKD stages were calculated as follows: (1) CKD stage was assessed based on GFR level; (2) GFR was retained downward for CKD stage 3 or later; and (3) the treatment effect on serum creatinine levels was assumed to be similar in patients with CKD stages 3 and 4. According to a previous study, the annual GFR decline rate was 1.7 mL/min/1.73 m^2^ (33), and the GFR level after treatment was estimated using the Modification of Diet in Renal Disease equation according to the characteristics of the Chinese population (Equation 1) (34). Changes in serum creatinine levels after treatment were calculated using the pooled effect estimate comparing Bailing capsules combined with conventional therapy versus conventional therapy alone. The microsimulation generated a cohort of 10,000 patients. The distributions of age, sex, serum creatinine levels at various CKD stages, and annual reductions in GFR are presented in Supplementary Table 1. Equation 1:


$$eGFR=186\times Scr^{-1.154}\times {\left(age\right)}^{-0.203}\left[\times 0.742\left(female\right)\right]$$

[×1.233(Chinesepopulation)]

Mortality rates for patients with CKD at various stages were determined based on previously published articles. The cost per cycle of Bailing capsules was determined as follows:

Bailing capsules per cycle cost = unit price of drug × daily dosage × cycle × medication compliance.

The price of Bailing capsules was CNY 43.26, containing 42 tablets (0.5 g/tablet). According to the drug label, the daily dose of Bailing capsules was 12 tablets. Medication compliance was obtained from a previous study, which reported a compliance rate of 92.75% (35). The costs of conventional therapy and hemodialysis were obtained from published studies. Utility values for each stage were based on a published study (36) that applied the Health Utilities Index Mark 3 scale. Details of the Markov model parameters are listed in Supplementary Table 2.

#### Sensitivity analysis

One-way and probabilistic sensitivity analyses (PSA) of the model parameters were performed to assess the robustness of the evaluation model. Results of the one-way sensitivity analysis were presented using a tornado diagram. The range of each parameter used for the one-way sensitivity analysis was based on either the 95% confidence interval (CI) reported in the referenced literature or a ± 30% change from the base-case value.

In the PSA, all costs were assigned a gamma distribution, while probabilities, proportions, and utilities were assigned beta distributions. Values were extracted from the corresponding distribution for 1,000 Monte Carlo simulations. The PSA results were presented using a cost-effectiveness acceptability curve.

## Results

### Meta-analysis

#### Literature search and study characteristics

**FIGURE 1:**
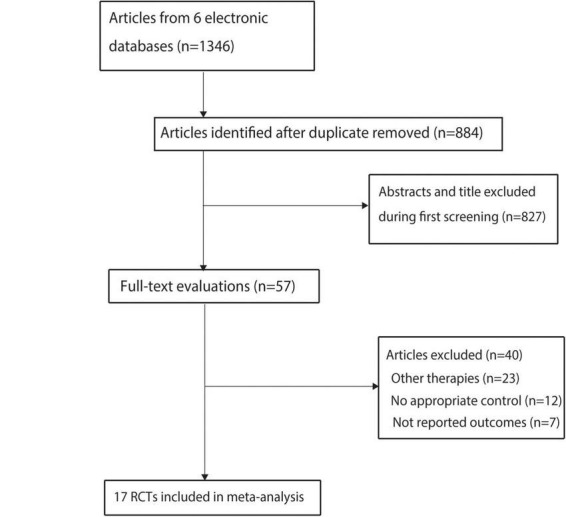
The flowchart regarding the study screening and selection.

The initial search yielded 1,346 articles, and 884 were retained after duplicates were removed. Subsequently, 827 studies were removed during title and abstract screening, and the remaining 57 articles were retrieved for full-text evaluation. Thereafter, 40 articles were removed because of other therapies (*n* = 23), lack of appropriate control (*n* = 12), or absence of reported serum creatinine levels (*n* = 7). The remaining 17 RCTs were selected for the final meta-analysis (Figure 1) (37–53).

The baseline characteristics of the included studies are presented in Table 1. A total of 1,380 patients were included, with treatment duration ranging from 2 weeks to 1 year. The methodological quality of the included trials is shown in Table 2. Overall, all the included studies were assessed as having a moderate risk of bias (ROB) according to the ROB2 tool.

**TABLE 2:** Quality assessment of included trials.

Study	Bias 1	Bias 2	Bias 3	Bias 4	Bias 5	Overall risk of bias
Zheng et al. (37)	Moderate	Moderate	Low risk	Low risk	Moderate	Moderate risk
Pu (38)	Moderate	Moderate	Low risk	Low risk	Moderate	Moderate risk
Zheng and Wang (39)	Moderate	Moderate	Low risk	Low risk	Moderate	Moderate risk
Xiong et al. (40)	Moderate	Moderate	Low risk	Low risk	Moderate	Moderate risk
Zhang and Wang (41)	Low risk	Moderate	Low risk	Low risk	Moderate	Moderate risk
Zheng et al. (42)	Low risk	Moderate	Low risk	Low risk	Moderate	Moderate risk
Meng and Guo (43)	Low risk	Moderate	Low risk	Low risk	Moderate	Moderate risk
Ru (44)	Moderate	Moderate	Low risk	Low risk	Moderate	Moderate risk
Huang et al. (45)	Low risk	Moderate	Low risk	Low risk	Moderate	Moderate risk
Yi (46)	Moderate	Moderate	Low risk	Low risk	Moderate	Moderate risk
He (47)	Moderate	Moderate	Low risk	Low risk	Moderate	Moderate risk
Jiang (48)	Low risk	Moderate	Low risk	Low risk	Moderate	Moderate risk
Shen and Zhang (49)	Moderate	Moderate	Low risk	Low risk	Moderate	Moderate risk
Pan (50)	Moderate	Moderate	Low risk	Low risk	Moderate	Moderate risk
Wang and Jiang (51)	Moderate	Moderate	Low risk	Low risk	Moderate	Moderate risk
Hu (52)	Low risk	Moderate	Low risk	Low risk	Moderate	Moderate risk
Ren (53)	Low risk	Moderate	Low risk	Low risk	Moderate	Moderate risk

#### Efficacy and safety

**FIGURE 2:**
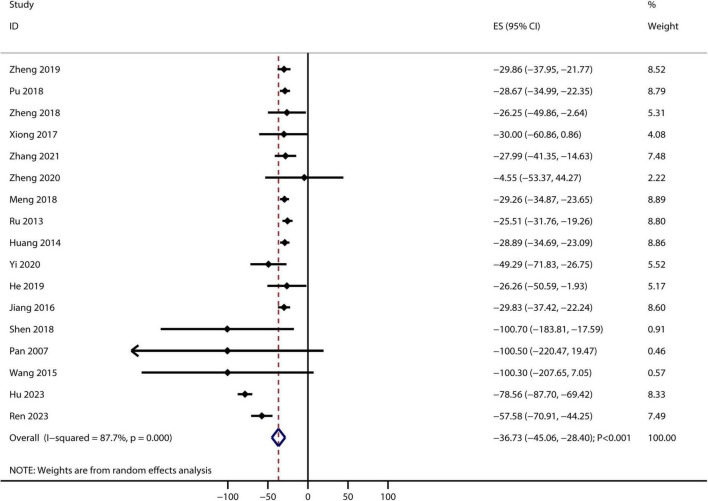
Bailing capsule plus conventional therapies versus conventional therapy alone on serum creatinine level. Forest plot showing pooled weighted mean differences (WMD) and 95% confidence intervals (CIs) for serum creatinine. Squares represent individual study effects, with size proportional to study weight; diamonds indicate the pooled result.

After pooling the included trials, significant heterogeneity was observed across them; therefore, a random-effects model was applied. Bailing capsules combined with conventional therapies were associated with a greater reduction in serum creatinine levels than conventional therapy alone (weighted mean difference [WMD]: −36.73 μmol/L; 95% CI: −45.06 to −28.40; *p* < 0.001; Figure 2). Significant heterogeneity was observed among the included studies (*I^2^* = 87.7%, *p* < 0.001). Sensitivity analysis indicated that the pooled conclusion was robust and not altered by excluding any particular trial (Supplementary Figure 2). Subgroup analyses revealed that Bailing capsules combined with conventional therapies were associated with a greater reduction in serum creatinine levels than conventional therapy alone in all subgroups (Supplementary Table 3). Moreover, potential significant publication bias for serum creatinine levels was detected (Egger’s test: *p* = 0.315; Begg’s test: *p* = 0.012; Supplementary Figure 3). However, the results remained stable after adjusting for potential publication bias using the trim-and-fill method.

**FIGURE 3:**
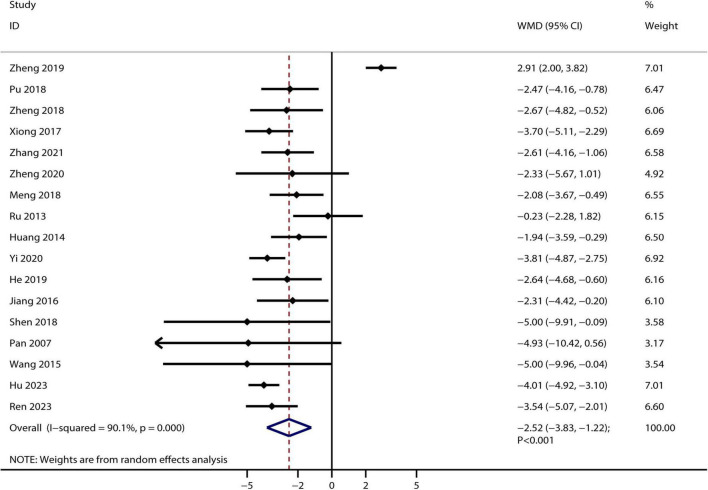
Bailing capsule combined with conventional therapies versus conventional therapy alone on blood urea nitrogen.

A total of 17 RCTs reported the therapeutic effect of Bailing capsules on BUN, and the summary result indicated that Bailing capsules combined with conventional therapies were associated with lower BUN than conventional therapy alone (WMD: −2.52 mmol/L; 95% CI: −3.83 to −1.22; *p* < 0.001; Figure 3). Moreover, significant heterogeneity was observed among the included studies (*I^2^* = 90.1%, *p* < 0.001).

**FIGURE 4:**
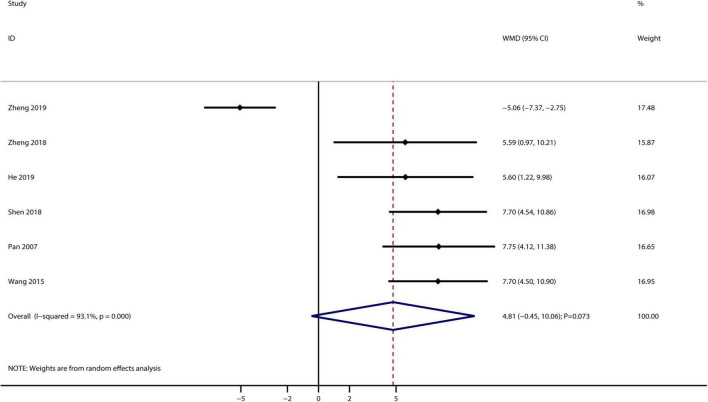
Bailing capsule combined with conventional therapies versus conventional therapy alone on creatinine clearance rate.

A total of six studies reported the therapeutic effect of Bailing capsules on Ccr, and no significant difference was observed between Bailing capsules combined with conventional therapies and conventional therapy alone for Ccr (WMD: 4.81 mL/min; 95% CI: −0.45–10.06; *p* = 0.073; Figure 4). Furthermore, significant heterogeneity was observed across the included studies (*I^2^* = 93.1%, *p* < 0.001).

**FIGURE 5:**
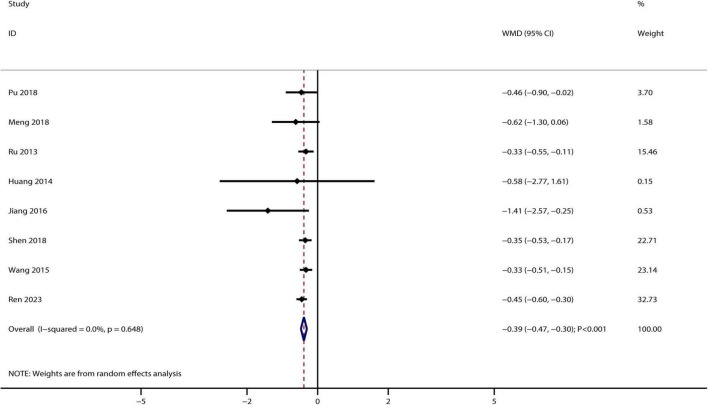
Bailing capsule combined with conventional therapies versus conventional therapy alone on 24 h urinary protein.

A total of eight studies reported the therapeutic effect of Bailing capsules on 24 h urinary protein, and the summary result indicated that Bailing capsules combined with conventional therapies were associated with lower 24 h urinary protein levels than conventional therapy alone (WMD: −0.39 g/L; 95% CI: −0.47, −0.30; *p* < 0.001; Figure 5). No evidence of heterogeneity was observed among the included studies (*I^2^* = 0.0%, *p* = 0.648).

The GRADE assessment revealed moderate-quality evidence for serum creatinine, BUN, and 24 h urinary protein, limited by high heterogeneity or a moderate risk of bias. Evidence for Ccr was rated as low quality due to sparse data in the included studies.

Only three of the included trials reported adverse events. Huang et al. reported two cases of pain at the injection site in both the Bailing capsule and control groups (44). Yi et al. reported one case each of throat discomfort, dizziness, and angialgia in patients treated with Bailing capsules, while the control group reported one case of throat discomfort, two cases of dizziness, and one case of diarrhea (45). Jiang et al. reported one case of throat discomfort and one case of nausea in patients treated with Bailing capsules, while the control group reported three cases of throat discomfort, two cases of nausea, and one case of vomiting (47).

### Economic evaluation

#### Base-case analysis

The QALYs in the Bailing capsule group and the conventional therapy group were 4.96 and 4.04, respectively. The use of Bailing capsules combined with conventional therapies was associated with an increase of 0.92 QALYs. Moreover, the costs for the Bailing capsule and conventional therapy groups were CNY 323,370 and CNY 302,035, respectively. The cost in the Bailing capsule group increased by CNY 21,335. The ICER was CNY 23,312 per QALY. The ICER was lower than the WTP (CNY 85,698), suggesting that the use of Bailing capsules for CRF is a cost-effective treatment option in the Chinese population.

#### Sensitivity analysis

**FIGURE 6:**
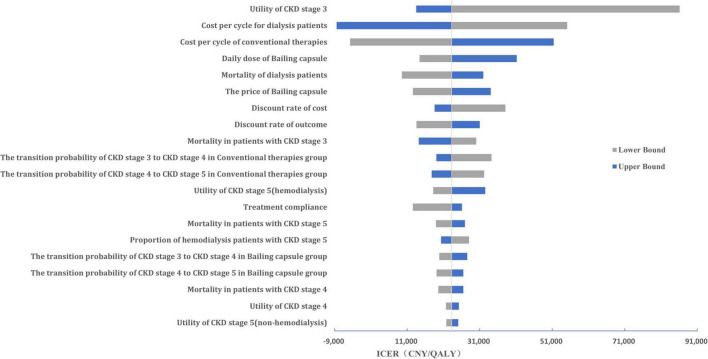
The Tornado diagram of the one-way sensitivity analysis. Key variables influencing the incremental cost-effectiveness ratio (ICER) are ranked by their impact, with bars indicating the range of ICER changes from worst- to best-case scenarios. QALY, quality-adjusted life year.

**FIGURE 7:**
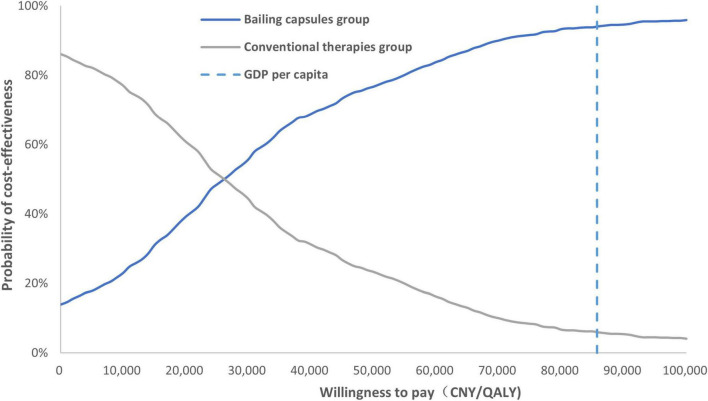
The cost-effectiveness acceptability curve. Probability that Bailing capsule plus conventional therapy is cost-effective at varying willingness-to-pay thresholds (CNY per QALY gained).

A tornado diagram of the one-way sensitivity analysis is shown in Figure 6. The important factors influencing the ICER included the CKD stage 3 utility value, hemodialysis cost per cycle, cost of conventional therapy, and daily dose of the Bailing capsule. Additionally, the cost-effectiveness acceptability curve indicated that the use of Bailing capsules combined with conventional therapies had a 94.0% probability of being cost-effective when the WTP threshold was CNY 85,698 (Figure 7).

## Discussion

Chronic renal failure is a gradually progressive outcome induced by various renal diseases that persist despite treatment. Without appropriate treatment, CRF can progress to uremia, resulting in severe renal dysfunction. Therefore, effective treatments are needed to slow the progression of CRF. Several studies have demonstrated the efficacy and safety of Bailing capsules in patients with CRF (13, 14); however, the cost-effectiveness of their use remains unclear. The current meta-analysis identified 17 RCTs and involved 1,380 patients with CRF. Our study found that Bailing capsules combined with conventional therapies were associated with a greater reduction in serum creatinine levels than conventional therapies alone. Moreover, Bailing capsules combined with conventional therapies were associated with lower BUN and 24 h urinary protein levels than conventional therapy alone, while no significant difference was observed between the groups in terms of changes in Ccr. Furthermore, the use of Bailing capsules combined with conventional therapies increased the QALYs by 0.92, and the cost increased by CNY 21,335. Finally, we noted that the use of Bailing capsules combined with conventional therapies had a 94.0% probability of being cost-effective when the WTP value was set at the 2022 GDP per capita (CNY 85,698). However, the low methodological quality of the included studies introduced potential bias and undermined the robustness of our conclusions. The reliance of our study on low-quality RCTs underscores methodological challenges in the field. Future studies should prioritize rigorous trial designs to generate credible evidence.

Several recent meta-analyses have evaluated TCM interventions for CKD, providing a basis for comparing our findings. A 2023 study by Song et al. assessed Niaoduqing granules for diabetic kidney disease, reporting a mean reduction in serum creatinine, BUN, and urinary albumin excretion rate alongside increased CCR with combined therapy—consistent with our observations for Bailing capsules (54). Another study evaluating rhubarb for CRF found significant reductions in serum creatinine and BUN, with increased CCR levels, comparable to our Bailing capsule results (4). However, these studies focused solely on clinical efficacy and omitted economic evaluations. Notably, a 2023 meta-analysis by Yin et al. found Uremic Clearance Granules significantly reduced serum creatinine levels while increasing GFR. While most TCM studies assess composite formulas, Bailing capsules contain a standardized *Cordyceps* mycelia extract, potentially enhancing reproducibility and mechanistic clarity compared to multi-herb decoctions (16). Important differences in study populations and interventions should be acknowledged. For instance, Long et al.’s study focused specifically on diabetic kidney disease (55), whereas our analysis includes non-diabetic CRF patients, limiting direct comparability. Future head-to-head studies are needed to explicitly compare the efficacy and cost-effectiveness of Bailing capsules with other TCM modalities.

The Bailing capsule contains bioactive components such as polysaccharides, adenosine, and cordycepin, which may exert renoprotective effects through multiple signaling pathways in CKD (56, 57). Dysregulation of transforming growth factor-beta (TGF-β)/Smad signaling is a key driver of renal fibrosis, promoting extracellular matrix accumulation and tubular epithelial-mesenchymal transition. Preclinical studies suggest that cordycepin, a major component of Bailing capsules, may inhibit TGF-β1-induced Smad2/3 phosphorylation, thereby reducing collagen deposition in renal tissues (58). Furthermore, Bailing capsules have been shown to downregulate angiotensin-converting enzyme activity, suppress the renin-angiotensin system (RAS) cascade, and improve glomerular hyperfiltration (59). This effect may intersect with the Wnt/β-catenin pathway, as RAS activation often upregulates Wnt1, leading to β-catenin-mediated podocyte injury, as previously demonstrated in sirtuin 6–based interventions that protect against podocyte injury by blocking the RAS-Wnt1/β-catenin axis (60). Additionally, the anti-hypoxic and anti-inflammatory properties of Bailing capsules may modulate nuclear factor-κB and mitogen-activated protein kinase pathways, reducing the production of pro-inflammatory cytokines and reactive oxygen species (61). These effects are consistent with studies showing that *Cordyceps* mycelia suppress the NOD-like receptor family pyrin domain-containing 3 inflammasome activation, which is a key driver of CKD progression. While direct mechanistic studies of Bailing capsules on these pathways are limited, the observed clinical benefits in serum creatinine reduction and CKD progression delay are consistent with the hypothesized modulation of TGF-β/Smad, RAS, and Wnt/β-catenin signaling. Future *in vitro* and *in vivo* studies are warranted to validate these mechanisms and clarify the specific components of Bailing capsules responsible for pathway regulation.

In addition to traditional renal signaling pathways, recent studies have highlighted the critical role of intestinal flora dysbiosis and metabolic disorders in CKD progression, offering novel therapeutic targets. Altered gut microbiota composition has been linked to the transition from acute kidney injury to CKD in aged mice. Dysbiosis promotes systemic inflammation and uremic toxin production, thereby exacerbating renal fibrosis (62). Probiotic interventions such as *Lactobacillus johnsonii* have shown promise in reversing CKD-associated gut dysbiosis and reducing serum creatinine levels in preclinical models (63). Notably, Bailing capsules contain bioactive compounds that may modulate gut microbiota diversity, analogous to prebiotics, by promoting beneficial bacterial growth and inhibiting pro-inflammatory species (64). Furthermore, in patients on peritoneal dialysis, gut-derived uremic toxins contribute to cardiovascular complications, which are a major cause of mortality in patients with CKD (65). The antioxidant properties of Bailing capsules may mitigate toxin-induced endothelial dysfunction; however, direct evidence is lacking. Dysregulation of phosphatidylcholine metabolism via phospholipase A2 has been observed in CKD rats, leading to the accumulation of profibrotic lysophosphatidylcholine (66). The adenosine component of the Bailing capsules may regulate lipid metabolic enzymes and potentially alter this pathway. Finally, in immunoglobulin A (IgA) nephropathy, gut microbiome–induced toll-like receptor 4 signaling promotes the production of hypoglycosylated IgA1, a key driver of glomerular injury (67). The immunomodulatory effects of Bailing capsules may disrupt the gut-kidney axis, although mechanistic validation is required.

This study found that Bailing capsules combined with conventional therapies were more cost-effective than conventional therapies alone. The pooled results for the effect of Bailing capsules combined with conventional therapies were stable, and the conclusion regarding cost-effectiveness was robust. The ICER for Bailing capsules was CNY 23,312/QALY, which is significantly lower than the WTP threshold. Moreover, one-way sensitivity analysis indicated that CKD stage 3 utility value, hemodialysis cost per cycle, cost of conventional therapy, and daily dose of bailing capsules were the most important factors affecting the cost-effectiveness of Bailing capsules. Furthermore, 94.0% of the patients were likely to accept the use of Bailing capsules combined with conventional therapies if the WTP was CNY 85,698. The price of the Bailing capsules and the WTP threshold value play an important role in determining the cost-effectiveness of the Bailing capsules. Considering that no evidence of heterogeneity was observed among the included trials, the treatment effectiveness of the Bailing capsules was stable. However, various disease statuses can affect the treatment effectiveness of Bailing capsules, and future context-specific studies on the primary economic evaluation of Bailing capsules are required.

The findings of this meta-analysis and the economic evaluation have direct clinical implications for CKD management in China. The 36.73 μmol/L reduction in serum creatinine observed with Bailing capsules aligns with clinically meaningful improvements in renal function for non-dialysis patients with CKD (stages 3–4), where even modest declines in creatinine can delay progression to dialysis. This is particularly relevant in resource-limited settings, where access to advanced therapies, such as renin-angiotensin system blockers, may be suboptimal. Additionally, the low ICER (CNY 23,312/QALY) suggests that Bailing capsules can be integrated into standard care at a reasonable cost, potentially reducing the healthcare burden associated with CKD progression.

Some limitations of this study include: (1) Treatment effectiveness based on meta-analysis, which has inherent limitations that should be examined cautiously, including inevitable publication bias and restricted detailed analyses; (2) Our study hypothesized that the treatment effect of the Bailing capsule mainly delayed disease progression in CKD stages 3 and 4, while several studies found that Bailing capsules could improve micro-inflammation and nutritional status in patients undergoing hemodialysis; therefore, the economic value of the Bailing capsule may be underestimated (68, 69); (3) Transition probabilities were calculated based on a short-term study using microsimulation as long-term follow-up trials in the Chinese population were not available; (4) Although we conducted extensive searches in international databases such as PubMed and Embase, no additional eligible English-language studies were found. This limits the generalizability of our findings to non-Chinese populations and healthcare systems. Future research should conduct more international, multicenter RCTs to validate further the efficacy and cost-effectiveness of Bailing capsules for CRF. (5) The high heterogeneity in our meta-analysis highlights variability in treatment responses across studies, potentially driven by differences in intervention duration, patient baseline characteristics, or concomitant medications. Although sensitivity and subgroup analyses were performed, the sources of heterogeneity were not fully explained. (6) Lack of data on additional clinical outcomes (e.g., GFR, albumin, and hemoglobin) in the included RCTs limited the depth of our analysis because these metrics are critical for assessing overall renal and nutritional status; (7) CRF encompasses a wide spectrum of CKD severity (stages 3–5), and treatment responses may vary across stages. Our inability to perform subgroup analysis by stage prevented precise recommendations for early versus advanced CRF. Future studies must collect and report stage-stratified outcomes to address this gap and guide personalized therapy; (8) the moderate risk of bias in all the included RCTs is a major limitation. However, because all studies shared similar methodological flaws and no trial was rated as “low risk,” a subgroup analysis excluding low-quality trials was not meaningful because the entire dataset lacked high-quality evidence. This uniformity in bias risk suggests that our pooled estimates may be directionally valid but with uncertain precision; and (9) the utilities in the Markov model and mean reduction of GFR per year were derived from studies conducted in other countries, which could affect the reliability of the cost-effectiveness of the Bailing capsule.

## Conclusion

This study indicated that the Bailing capsule combined with conventional therapies was associated with a greater reduction in serum creatinine levels and was more cost-effective than conventional therapies alone for treating CRF. Although our findings suggest the potential benefits of the Bailing capsule, they are constrained by the low methodological quality of the included studies. High-quality, well-conducted RCTs are essential to validate these results and ensure their clinical applicability. Moreover, further long-term follow-up trials should be performed to verify the cost-effectiveness of Bailing capsules in patients with CRF.

## Data Availability

The original contributions presented in this study are included in this article/Supplementary material, further inquiries can be directed to the corresponding author.

## Supplementary material

Supplementary Figure 1 Markov model.

Supplementary Figure 2 Sensitivity analysis for serum creatinine.

Supplementary Figure 3 Funnel plot for serum creatinine.

## References

[1] Martínez-Hernández S Muñoz-Ortega M Ávila-Blanco M Medina-Pizaño M Ventura-Juárez J. Novel approaches in chronic renal failure without renal replacement therapy: A review. *Biomedicines.* (2023). 11:2828. 10.3390/biomedicines11102828 37893201 PMC10604533

[2] Terzo C Gembillo G Cernaro V Longhitano E Calabrese V Casuscelli C Investigational new drugs for the treatment of chronic renal failure: An overview of the literature. *Expert Opin Investig Drugs.* (2024) 33:319–34. 10.1080/13543784.2024.2326624 38429874

[3] Wang Y Yang J Zhang Y Zhou J. Focus on mitochondrial respiratory chain: Potential therapeutic target for chronic renal failure. *Int J Mol Sci.* (2024) 25:949. 10.3390/ijms25020949 38256023 PMC10815764

[4] Huang W Rao Y Li L Li C An Y. Clinical effect of rhubarb on the treatment of chronic renal failure: A meta-analysis. *Front Pharmacol.* (2023) 14:1108861. 10.3389/fphar.2023.1108861 37153797 PMC10157189

[5] Xie F Zhang T Zhang P Qu X Li M Lan W. Shenkang injection combined with alprostadil for chronic renal failure: A systematic review and meta-analysis. *Front Med.* (2023) 10:982016. 10.3389/fmed.2023.982016 37089596 PMC10118024

[6] Skalsky K Shiyovich A Steinmetz T Kornowski R. Chronic renal failure and cardiovascular disease: A comprehensive appraisal. *J Clin Med.* (2022) 11:1335. 10.3390/jcm11051335 35268426 PMC8911484

[7] Gronda E Vanoli E Iacoviello M Urbinati S Caldarola P Colivicchi F Renal effects of SGLT2 inhibitors in cardiovascular patients with and without chronic kidney disease: Focus on heart failure and renal outcomes. *Heart Fail Rev.* (2023) 28:723–32. 10.1007/s10741-021-10211-9 35098383 PMC8801273

[8] Wang W Zhang X Yin H Li X Hu X Liu H Effects of Bailing capsules for renal transplant recipients: A retrospective clinical study. *Chin Med J.* (2013) 126:1895–9. 10.3760/cma.j.issn.0366-6999.20130483 23673106

[9] Hu X Wang J Yang H Ji S Li Y Xu B Bailing Capsule combined with α-ketoacid tablets for stage 3 chronic kidney disease: Protocol of a double-blinded, randomized, controlled trial. *Medicine.* (2021) 100:e25759. 10.1097/md.0000000000025759 34011035 PMC8136994

[10] Zhou X Ye J Guo X Chen M. Therapeutic effect of Corbrin (Bailing) capsule on patients with renal insufficiency: A meta-analysis. *Heliyon.* (2024) 10:e29488. 10.1016/j.heliyon.2024.e29488 38699752 PMC11063392

[11] He Y Li W Zhu H Han S. Economic evaluation of bailing capsules for patients with diabetic nephropathy in China. *Front Pharmacol.* (2023) 14:1175310. 10.3389/fphar.2023.1175310 37475712 PMC10354420

[12] Zhang Q Xiao X Li M Yu M Ping F. Bailing capsule (Cordyceps sinensis) ameliorates renal triglyceride accumulation through the PPARα pathway in diabetic rats. *Front Pharmacol.* (2022) 13:915592. 10.3389/fphar.2022.915592 36091833 PMC9453879

[13] Liu Z Zhang Y Hu X. Systematic evaluation of efficacy of corbrin capsule for chronic renal failure. *Clin Med J.* (2017) 15:37–42. 10.21037/apm-22-291 35523751

[14] Zhang R Cai H Hu R. Analysis of symptom improvement of chronic renal failure treated with Bailing capsule. *Healthful Friend.* (2020) 2:104. 10.1097/MD.0000000000025759 34011035 PMC8136994

[15] Zheng S Xu Y Zhang Y Long C Chen G Jin Z Efficacy and safety of traditional Chinese medicine decoction as an adjuvant treatment for diabetic nephropathy: A systematic review and meta-analysis of randomized controlled trials. *Front Pharmacol.* (2024) 15:1327030. 10.3389/fphar.2024.1327030 38783937 PMC11111926

[16] Yin J Chen H Zhu B. The safety and efficacy of using uremic clearance granules for treating stages 3 to 5 of chronic kidney disease: A meta-analysis. *Integr Med Nephrol Androl.* (2023) 10:e00013. 10.1097/IMNA-D-23-00013

[17] Wu F Xu C Si X He F Xu K Zhang Y Efficacy of traditional Chinese medicine Cordyceps sinensis as an adjunctive treatment in patients with renal dysfunction: A systematic-review and meta-analysis. *Front Med.* (2025) 11:1477569. 10.3389/fmed.2024.1477569 39839641 PMC11747039

[18] Tao Y Luo R Xiang Y Lei M Peng X Hu Y. Use of bailing capsules (cordyceps sinensis) in the treatment of chronic kidney disease: A meta-analysis and network pharmacology. *Front Pharmacol.* (2024) 15:1342831. 10.3389/fphar.2024.1342831 38645562 PMC11026558

[19] Page M McKenzie J Bossuyt P Boutron I Hoffmann T Mulrow C The PRISMA 2020 statement: An updated guideline for reporting systematic reviews. *Bmj.* (2021) 372:n71. 10.1136/bmj.n71 33782057 PMC8005924

[20] Flemyng E Moore T Boutron I Higgins J Hróbjartsson A Nejstgaard C Using risk of Bias 2 to assess results from randomised controlled trials: Guidance from Cochrane. *BMJ Evid Based Med*. (2023) 28:260–6. 10.1136/bmjebm-2022-112102 36693715

[21] Chu D Golden D Guyatt G. Translating evidence to optimize patient care using GRADE. *J Allergy Clin Immunol Pract.* (2021) 9:4221–30. 10.1016/j.jaip.2021.09.035 34624540

[22] Higgins J Thompson S Deeks J Altman D. Measuring inconsistency in meta-analyses. *Bmj.* (2003) 327:557–60. 10.1136/bmj.327.7414.557 12958120 PMC192859

[23] DerSimonian R Laird N. Meta-analysis in clinical trials revisited. *Contemp Clin Trials.* (2015) 45(Pt A):139–45. 10.1016/j.cct.2015.09.002 26343745 PMC4639420

[24] Meng Z Wang J Lin L Wu C. Sensitivity analysis with iterative outlier detection for systematic reviews and meta-analyses. *Stat Med.* (2024) 43:1549–63. 10.1002/sim.10008 38318993 PMC10947935

[25] Maier M VanderWeele T Mathur M. Using selection models to assess sensitivity to publication bias: A tutorial and call for more routine use. *Campbell Syst Rev.* (2022) 18:e1256. 10.1002/cl2.1256 36909879 PMC9247867

[26] Almalik O Zhan Z van den Heuvel E. Tests for publication bias are unreliable in case of heteroscedasticity. *Contemp Clin Trials Commun.* (2021) 22:100781. 10.1016/j.conctc.2021.100781 34179565 PMC8209747

[27] Zhang L Zuo L. Current burden of end-stage kidney disease and its future trend in China. *Clin Nephrol.* (2016) 86:27–8. 10.5414/cnp86s104 27469147

[28] Liu G. *Evaluation Guide of Chinese Pharmacoeconomics.* Beijing: China Market Press (2020).

[29] Lai X Zhang A Chen S He L Su C Fan M Outcomes of stage 1-5 chronic kidney disease in Mainland China. *Ren Fail.* (2014) 36:520–5. 10.3109/0886022x.2013.875859 24456114

[30] Zhang L Wang F Wang L Wang W Liu B Liu J Prevalence of chronic kidney disease in China: A cross-sectional survey. *Lancet.* (2012) 379:815–22. 10.1016/s0140-6736(12)60033-6 22386035

[31] Weng S Tarng D Chen C Cheng C Wu M Chen C Estimated glomerular filtration rate decline is a better risk factor for outcomes of systemic disease-related nephropathy than for outcomes of primary renal diseases. *PLoS One.* (2014) 9:e92881. 10.1371/journal.pone.0092881 24695125 PMC3973643

[32] Guan H Han S Wang Y. Pharmacoeconomic evaluation of Shenyankangfu tablets plus conventional therapy in treating diabetic nephropathy. *Chin J New Drugs.* (2017) 26:2491–6.

[33] Orlando L Belasco E Patel U Matchar D. The chronic kidney disease model: A general purpose model of disease progression and treatment. *BMC Med Inform Decis Mak.* (2011) 11:41. 10.1186/1472-6947-11-41 21679455 PMC3132702

[34] Chinese eGFR Investigation Collaboration. *MDRD.* New York, NY: National Kidney Foundation (2011).

[35] Chen W Mo Y Wang J. Investigation on medical compliance of patients with chronic kidney disease. *J PLA Nurs.* (2011) 28:8–10.

[36] Gorodetskaya I Zenios S McCulloch C Bostrom A Hsu C Bindman A Health-related quality of life and estimates of utility in chronic kidney disease. *Kidney Int.* (2005) 68:2801–8. 10.1111/j.1523-1755.2005.00752.x 16316356

[37] Zheng X Chen Z Deng Y. Effects of Bailing capsule on cellular immunity and renal function in patients with CKD stage 3-4 chronic renal failure. *Int J Urol Nephrol.* (2019) 39:1081–5. 10.3760/cma.j.issn.1673-4416.2019.06.033 30704229

[38] Pu Y. Clinical efficacy analysis of alprostadil combined with Bailing capsule in the treatment of chronic renal failure. *Inner Mongolia Tradit Chin Med.* (2018) 37:60–1. 10.16040/j.cnki.cn15-1101.2018.11.043

[39] Zheng J Wang X. Clinical analysis of Bailing capsule combined with compound α-ketoacid tablets in the treatment of chronic renal failure. *J Pract Tradit Chin Med.* (2018) 34:1230–1.

[40] Xiong Y Jiang B Tang J. Effect of Bailing capsule combined with Rhubarb Zechi pill on TGF-β1 and Col-IV in patients with chronic renal failure. *Chin J Integrat Tradit Western Nephrol.* (2024) 18:236–8.

[41] Zhang P Wang X. Effect of Bailing capsule on malnutrition and renal function in patients with chronic kidney disease in stage 3 and 4. *Shaanxi J Tradit Chin Med.* (2021) 42:54–6.

[42] Zheng X Chen Z Deng Y. Effect of Bailing capsule combined with α-Keto acid tablets on nutritional status and quality of life in patients with CKD 3-4 stage of chronic kidney disease. *J Tianjin Univ Tradit Chin Med.* (2020) 39:56–61.

[43] Meng Q Guo X. Analysis of therapeutic effect of alprostadil combined with Bailing capsule regimen on chronic renal failure. *J Psychiat.* (2018) 24:148–9.

[44] Ru Y. Effect of Bailing capsule on proteinuria in patients with chronic renal failure. *Chin J Med Guide.* (2013) 11:669–70. 10.15912/j.cnki.gocm.2013.12.458

[45] Huang L Wu J Liao T. Effect of alprostadil combined with Bailing capsule on chronic renal failure. *Chin J Pharmac.* (2014) 17:1162–4.

[46] Yi X. Effect analysis of Bailing capsule combined with α-ketoacid tablets on patients with chronic renal failure (CRF). *J Diabetes World.* (2020) 17:48.

[47] He M. Clinical effect analysis of Bailing capsule combined with compound α-ketoacid tablets on chronic renal failure. *Health Care Guide.* (2019) 47:94.

[48] Jiang N. Clinical effect of Bailing capsule on chronic renal failure. *Chin J Med Guide.* (2016) 14:190–1. 10.15912/j.cnki.gocm.2016.27.155

[49] Shen Y Zhang X. Effect of Bailing capsule on renal function and quality of life in patients with chronic renal failure. *Chin J Clin Rational Drug Use.* (2018) 11:77–8. 10.15887/j.cnki.13-1389/r.2018.22.042

[50] Pan H. Clinical observation of Bailing capsule in treatment of chronic renal failure. *Mod Med Heal.* (2007) 3:345–6. 10.1097/MD.0000000000025759 34011035 PMC8136994

[51] Wang J Jiang X. Effect of Bailing capsule on improving renal function and quality of life in patients with chronic renal failure. *J New Chin Med.* (2015) 47:65–7. 10.13457/j.cnki.jncm.2015.12.029

[52] Hu J. Effects of Bailing capsules combined with alprostadil injection on kidney function and micro-inflammatory state in patients with chronic renal failure. *J New Chin Med.* (2023) 55:73–7. 10.13457/j.cnki.jncm.2023.04.016

[53] Ren L. Observation of curative effect of Bailing capsule on chronic renal failure complicated with diabetes. *Women’s Health Res.* (2023) 9:118–20.

[54] Song C Zhu Z Liu L Liu S Li Y Xiao Y The efficacy and safety of Niaoduqing granules in the treatment of diabetic kidney disease: A systematic review and meta-analysis. *Front Pharmacol.* (2023) 14:1180751. 10.3389/fphar.2023.1180751 37475716 PMC10354524

[55] Long C Feng H Liu Z Li Z Liu J Jiang Y Efficacy of traditional Chinese medicine injection for diabetic kidney disease: A network meta analysis and systematic review. *Front Pharmacol.* (2023) 14:1028257. 10.3389/fphar.2023.1028257 36874023 PMC9981802

[56] Zhu L Zheng Z. Observation of clinical effect of integrated traditional Chinese and western medicine on nephrotic syndrome. *Med Forums Basic.* (2016) 20:4879–80.

[57] Zhang Y Xu L Lu Y Zhang J Yang M Tian Y Protective effect of Cordyceps sinensis against diabetic kidney disease through promoting proliferation and inhibiting apoptosis of renal proximal tubular cells. *BMC Complement Med Ther.* (2023) 23:109. 10.1186/s12906-023-03901-4 37024857 PMC10077712

[58] Salvadori M Rosso G. What is new in the pathogenesis and treatment of IgA glomerulonephritis. *World J Nephrol.* (2024) 13:98709. 10.5527/wjn.v13.i4.98709 39723359 PMC11572654

[59] Luo Y Yang S Zhou X Wang M Tang D Liu F Use of Ophiocordyceps sinensis (syn. Cordyceps sinensis) combined with angiotensin-converting enzyme inhibitors (ACEI)/angiotensin receptor blockers (ARB) versus ACEI/ARB alone in the treatment of diabetic kidney disease: A meta-analysis. *Ren Fail.* (2015) 37:614–34. 10.3109/0886022X.2015.1009820 25682973

[60] Miao H Wang Y Su W Zou L Zhuang S Yu X Sirtuin 6 protects against podocyte injury by blocking the renin-angiotensin system by inhibiting the Wnt1/β-catenin pathway. *Acta Pharmacol Sin.* (2024) 45:137–49. 10.1038/s41401-023-01148-w 37640899 PMC10770168

[61] Hattori T Fujioka K Nagai T Kondo S Kagami S Hirayama M Intrarenal renin-angiotensin system activation and macrophage infiltrations in pediatric chronic glomerulonephritis. *Pediatr Nephrol.* (2023) 38:3711–9. 10.1007/s00467-023-06026-5 37231123 PMC10514104

[62] Kim M Cho W Chung S Choi Y Fang Y Park M Altered gut microbiome plays an important role in AKI to CKD transition in aged mice. *Front Med.* (2023) 10:1238960. 10.3389/fmed.2023.1238960 38020091 PMC10644820

[63] Miao H Liu F Wang Y Yu X Zhuang S Guo Y Targeting *Lactobacillus johnsonii* to reverse chronic kidney disease. *Signal Transduct Target Ther.* (2024) 9:195. 10.1038/s41392-024-01913-1 39098923 PMC11298530

[64] Favero C Ortiz A Sanchez-Niño M. Probiotics for kidney disease. *Clin Kidney J*. (2022) 15:1981–6. 10.1093/ckj/sfac056 36325000 PMC9613434

[65] Donderski R Stróżecki P Sulikowska B Grajewska M Miśkowiec I Stefańska A Aldosterone antagonist therapy and its relationship with inflammation, fibrosis, thrombosis, mineral-bone disorder and cardiovascular complications in peritoneal dialysis (PD) patients. *Int Urol Nephrol.* (2017) 49:1867–73. 10.1007/s11255-017-1655-2 28710615 PMC5603618

[66] Wang Y Zhang Z Liu H Guo Z Zou L Zhang Y Integrative phosphatidylcholine metabolism through phospholipase A2 in rats with chronic kidney disease. *Acta Pharmacol Sin.* (2023) 44:393–405. 10.1038/s41401-022-00947-x 35922553 PMC9889763

[67] Zhu Y He H Sun W Wu J Xiao Y Peng Y IgA nephropathy: Gut microbiome regulates the production of hypoglycosilated IgA1 via the TLR4 signaling pathway. *Nephrol Dial Transplant.* (2024) 39:1624–41. 10.1093/ndt/gfae052 38402460 PMC11427068

[68] Zhu J. Effects of Bailing capsule on nutritional status and inflammatory cytokines in dialysis patients with chronic renal failure. *J New Chin Med.* (2019) 51:154–6. 10.13457/j.cnki.jncm.2019.08.046

[69] Jiang G Chi Z Qiu F. Effect of Bailing capsule on microinflammation and nutritional status in maintenance hemodialysis patients. *Chin Foreign Med Res.* (2020) 454:16–8. 10.19540/j.cnki.cjcmm.20211222.501 35531703

