# Supplementary figures and images for: Combined bailing capsule and conventional therapies in the treatment of chronic renal failure: a meta-analysis and economic evaluation
Source: Front Med (Lausanne). 2025 Jun 25;12:1609311. doi: 10.3389/fmed.2025.1609311 (PMC12238058; doi:10.3389/fmed.2025.1609311)

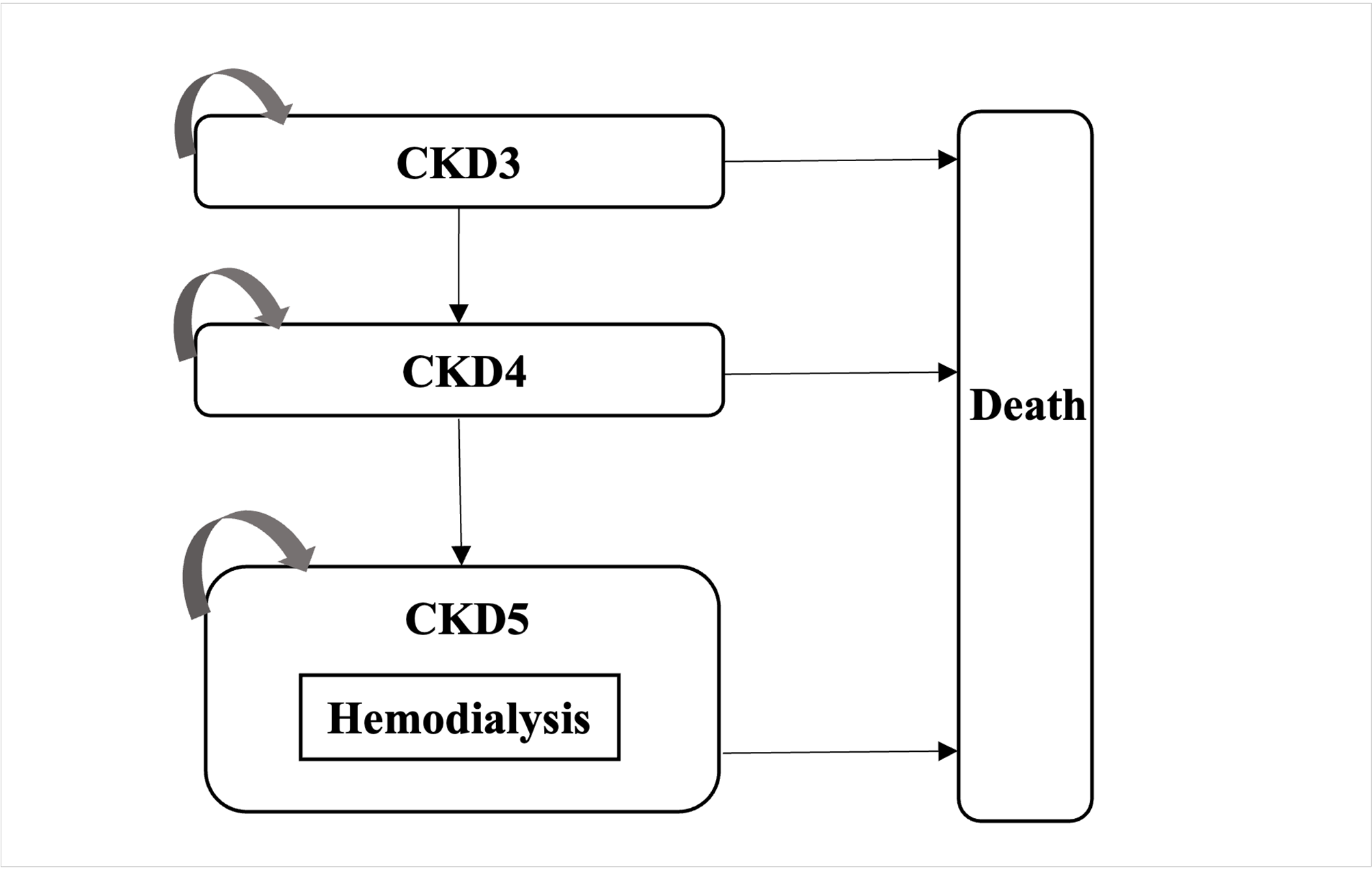

Supplement: Supplementary Figure 1 — Markov model. [file Image_1.tif]

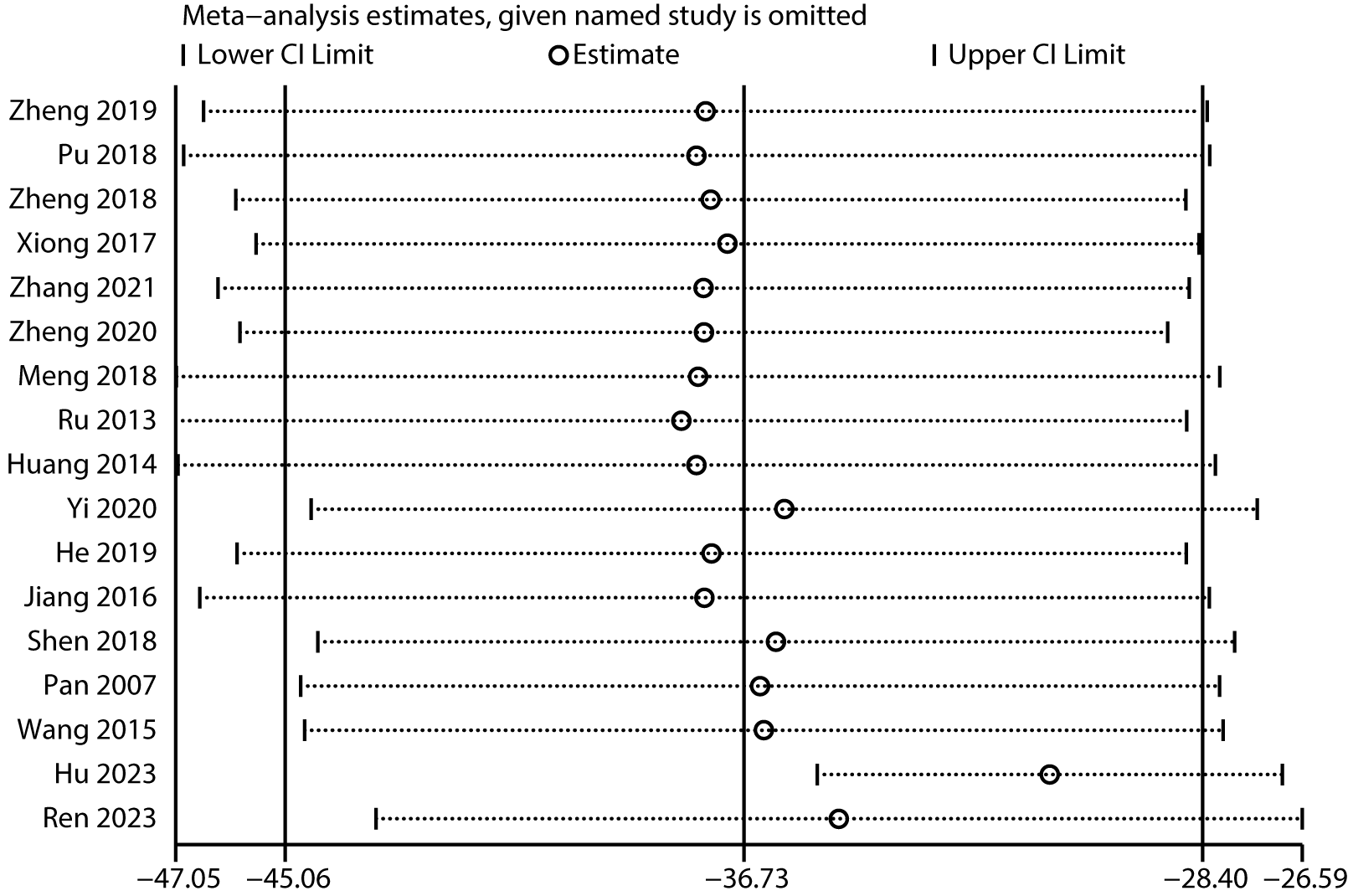

Supplement: Supplementary Figure 2 — Sensitivity analysis for serum creatinine. [file Image_2.tif]

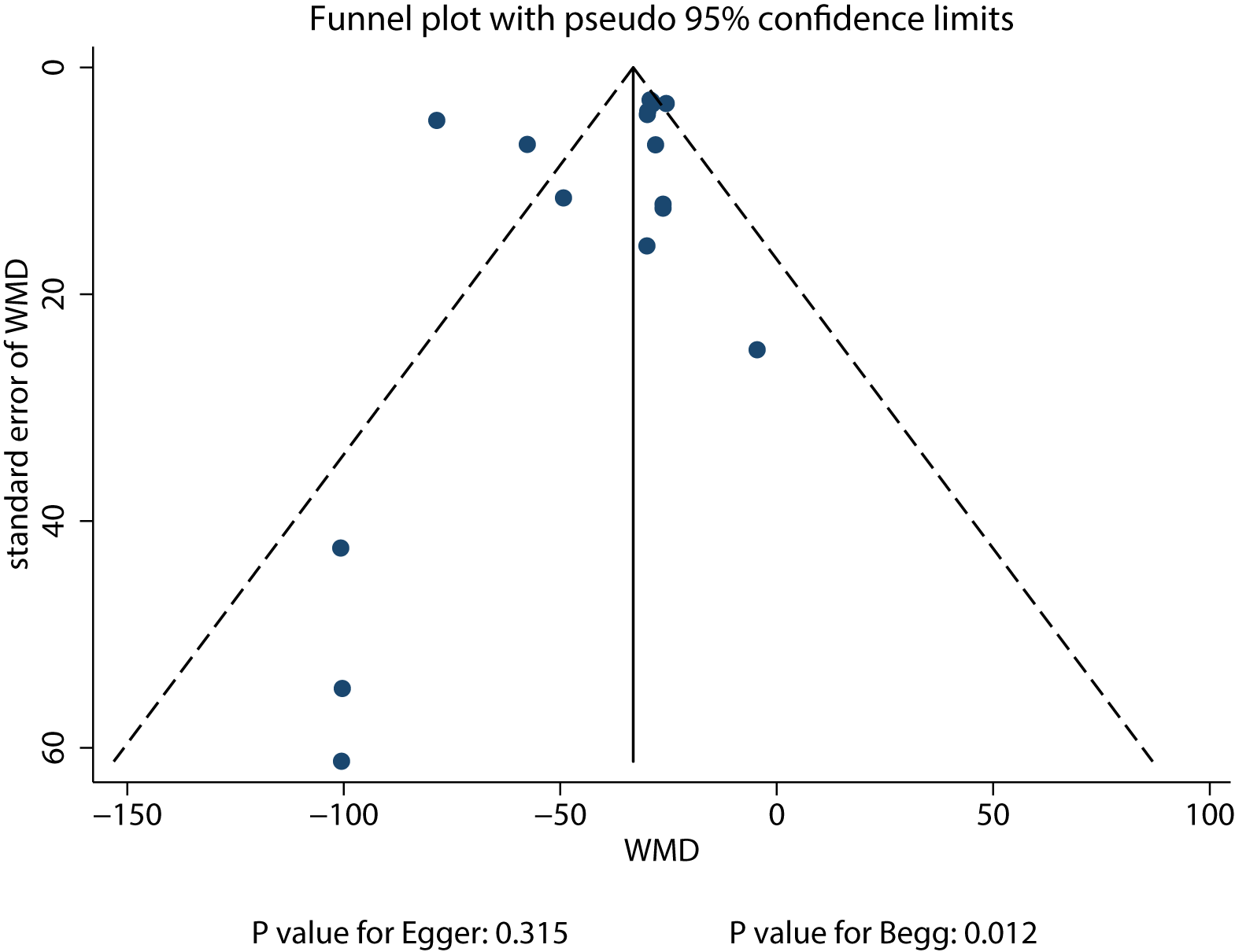

Supplement: Supplementary Figure 3 — Funnel plot for serum creatinine. [file Image_3.tif]
