# Supplementary material for: Combined bailing capsule and conventional therapies in the treatment of chronic renal failure: a meta-analysis and economic evaluation
Source: Front Med (Lausanne). 2025 Jun 25;12:1609311. doi: 10.3389/fmed.2025.1609311 (PMC12238058; doi:10.3389/fmed.2025.1609311)
Supplement: Supplementary file 4 [file Supplementary_file_1.docx]

**Search strategy in PubMed:**

#1 chronic renal failure [Title/Abstract]

#2 CRF [Title/Abstract]

#3 chronic kidney diseases [Title/Abstract]

#4 CKD [Title/Abstract]

#5 chronic kidney failure [Title/Abstract]

#6 #1 OR #2 OR #3 OR #4 OR #5

#7 corbrin capsule [Title/Abstract]

#8 aweto [Title/Abstract]

#9 artificial aweto preparation [Title/Abstract]

#10 cordyceps sinensis [Title/Abstract]

#11 Chinese caterpillar fungus [Title/Abstract]

#12 Chinese medicine [Title/Abstract]

#13 traditional Chinese medicine [Title/Abstract]

#14 combine traditional Chinese and western medicine [Title/Abstract]

#15 #7 OR #8 OR #9 OR #10 OR #11 OR #12 OR #13 OR #14

#16 clinical trial [Filter]

#17 randomized controlled trial [Filter]

#18 #16 OR #17

#19 #6 AND #15 AND #18
