# Supplementary material for: Combined bailing capsule and conventional therapies in the treatment of chronic renal failure: a meta-analysis and economic evaluation
Source: Front Med (Lausanne). 2025 Jun 25;12:1609311. doi: 10.3389/fmed.2025.1609311 (PMC12238058; doi:10.3389/fmed.2025.1609311)
Supplement: Supplementary file 7 [file Table_1.docx]

Table S1. Simulation parameters of microsimulation

| **Parameters** | **Range and distribution** | **Source** |
| --- | --- | --- |
| Age | 35-80 years, uniform distribution | Assumption |
| Sex | Male: female=1:1, uniform distribution | Assumption |
| CKD Stage 3 GFR | 30-59 ml/min/1.73 m^2^ | Reference [1] |
| CKD Stage 4 GFR | 15-29 ml/min/1.73 m^2^ | Reference [1] |
| CKD Stage 3 initial serum creatinine | 1.5-3.0 mg/dl, uniform distribution | Reference [2] |
| CKD Stage 4 initial serum creatinine | 3.1-5.0 mg/dl, uniform distribution | Reference [2] |
| The reduction of serum creatinine in control group | -0.349 mg/dl | Calculated* |
| Mean change of serum creatinine between intervention and control group | -0.415 mg/dl (95%CI：-0.510~-0.321), uniform distribution | Meta-analysis |
| Mean reduction of GFR per year | 1.7±3.4 ml/min/1.73 m^2^，normal distribution | Reference [3] |

*The reduction of serum creatinine in conventional therapies group from meta-analysis*the weight of individual trial in meta-analysis

**Reference:**

1. Chen XM, Ni ZH, Liu YN, et al. Guidelines for the diagnosis and treatment of chronic renal failure. CJITWM 2015; 35: 1029-1033.
2. Chinese Medical Association. Clinical diagnosis and treatment guidelines: Nephrology section. People’s Medical Publishing House, 2011.
3. Orlando LA, Belasco EJ, Patel UD, et al. The chronic kidney disease model: a general purpose model of disease progression and treatment. BMC Med Inform Decis Mak. 2011; 11: 41.
