# Supplementary material for: Combined bailing capsule and conventional therapies in the treatment of chronic renal failure: a meta-analysis and economic evaluation
Source: Front Med (Lausanne). 2025 Jun 25;12:1609311. doi: 10.3389/fmed.2025.1609311 (PMC12238058; doi:10.3389/fmed.2025.1609311)
Supplement: Supplementary file 9 [file Table_3.docx]

Table S3. Subgroup analysis for serum cretinine level

| Factors | Groups | No. of studies | WMD and 95%CI | *P* value | *I^2^* (%) | Q statistic |
| --- | --- | --- | --- | --- | --- | --- |
| Sample size | ≥ 100 | 5 | -29.17 (-33.67 to -24.67) | < 0.001 | 0.0 | 0.480 |
|  | < 100 | 12 | -39.69 (-51.59 to -27.78) | < 0.001 | 90.8 | < 0.001 |
| Mean age (years) | ≥ 50.0 | 8 | -41.95 (-60.07 to -23.84) | < 0.001 | 93.1 | < 0.001 |
|  | < 50.0 | 9 | -29.48 (-32.60 to -26.37) | < 0.001 | 3.6 | 0.405 |
| Disease duration (years) | ≥ 5.0 | 3 | -48.48 (-90.49 to -6.47) | 0.024 | 63.0 | 0.067 |
|  | < 5.0 | 8 | -37.98 (-51.58 to -24.38) | < 0.001 | 93.0 | < 0.001 |
| Treatment duration (months) | ≤ 3.0 | 13 | -37.44 (-47.02 to -27.86) | < 0.001 | 89.9 | < 0.001 |
|  | > 3.0 | 4 | -32.23 (-50.32 to -14.14) | < 0.001 | 36.8 | 0.191 |
